# Development of a colloidal gold immunochromatographic strip for the rapid detection of antibodies against *Fasciola gigantica* in buffalo

**DOI:** 10.3389/fvets.2022.1004932

**Published:** 2022-09-16

**Authors:** Jinhui Wang, Kangxin He, Zhengjiao Wu, Weikun Jin, Wende Wu, Yanfeng Guo, Weiyu Zhang, Wenda Di

**Affiliations:** ^1^College of Animal Science and Technology, Guangxi University, Nanning, China; ^2^Guangxi Zhuang Autonomous Region Engineering Research Center of Veterinary Biologics, Guangxi University, Nanning, China

**Keywords:** buffalo, colloidal gold, diagnosis, *Fasciola gigantica*, immunochromatographic

## Abstract

**Background:**

*Fasciola gigantica*, a tropical liver fluke, infects buffalo in Asian and African countries, causing significant economic losses and posing public health threats. The diagnostic of buffalo fascioliasis caused by *F. gigantica* is vital in fascioliasis control and preventation. The 22nd gel filtration chromatography fraction of *F. gigantica* Excretory-Secretory Products (*Fg*ESP), namely *Fasciola* 22 (F22), which was used as a diagnostic antigen in indirect ELISA, has demonstrated great potential for fascioliasis diagnosing. In the absence of rapid diagnostic methods, the use of a colloidal gold immunochromatographic strip based on F22 was applied to detect *F. gigantica* infection in buffalo.

**Methods:**

In the present study, the 22^nd^ gel filtration chromatography fraction of *Fg*ESP (F22) was used as an antigen to establish the colloidal gold-based immunochromatographic strip (ICS). The nitrocellulose membrane was incubated with F22 at the test line (T line) and goat anti-mouse secondary antibody at the control line (C line). The mouse anti-buffalo secondary antibody 2G7 conjugated to colloidal gold particles was used as the detection system for line visualization. The strip was assembled and developed by optimizing reaction conditions. The sensitivity, specificity, stability, and early diagnostic value of the strip were evaluated employing buffalo-derived sera.

**Results:**

An immunochromatographic strip for the rapid detection of antibodies against *F. gigantica*-*Fg*ICS was developed. The strip demonstrated high sensitivity and specificity. Sensitivity tests confirmed positive results even when the positive reference serum was diluted 4,096 times. Except for one *Schistosoma japonicum*-positive serum that tested positive via *Fg*ICS, specificity tests confirmed no cross-reactivity with other positive sera of *Schistosoma japonicum* and *Babesia bovis*. The strip remained stable after storage at 4°C for up to 3 months. In infected buffalo, antibodies could be detected as early as 14–21 days post-infection. The detection of 17 positive sera yielded an 82.4% positive rate via *Fg*ICS vs. a 100.0% positive rate via ELISA based on *Fg*ESP. For *Fg*ICS, the 95% confidence interval of sensitivity was 84.8–95.4%, while specificity was 4.2–14.7%.

**Conclusion:**

The immunochromatographic strip *Fg*ICS developed in this study provides a simple and rapid method of *F. gigantica* antibody detection and infected buffalo monitoring in the field.

## Introduction

Infection with *Fasciola* species, including *Fasciola hepatica* and *Fasciola gigantica* has been reported in a wide variety of mammalian species globally, including human, cattle, buffalo, and sheep, causing tremendous economic loss (1, 2). The epidemic of *F. hepatica* is mainly prevalent in temperate regions, while the epidemic of *F. gigantica* is mainly prevalent in tropical and subtropical regions (3). The World Health Organization estimates that 2.4 million people in more than 70 countries have been infected with *Fasciola*, and several millions people are at risk of infection (4). Compared with human, fasciolasis ruminants such as cattle, sheep and buffalo are also common globally. A report in Australia detected that the infection rate of *Fasciola* in sheep and cattle was 52.2 and 26.5%, respectively. In Asia, the infection rate of cattle was 0.7–69.2%, and that of goats was 0.0–47.0%, which suggested that *Fasciola* infection cause enormous economic losses to husbandry in various regions.

Buffalo are economically important animals in Asian and African countries, producing meat and milk but also serving as working animals in rural areas. In 2021, a survey, in which dissection was used for examining *F. gigantica* adult, suggested that all the flukes were *F. gigantica*, and no *Fasciola hepatica* or the intermediate form was found in Nanning, South of the China (5). Thus, *F. gigantica* infection in buffalo cannot be ignored. The development and improvement of diagnostic methods represent crucial countermeasures in preventing losses caused by *F. gigantica*. Given the low sensitivity and time-consuming process of coprological examination, serological examination is generally used in fascioliasis diagnosis (6, 7).

At present, serological analysis for *F. gigantica* mainly focuses on ELISA, which can be conducted through the detection of circulating antibodies or antigens (8). With ELISA, specific anti-*F. gigantica* antibodies can be detected as early as 2 weeks post-infection (pi) in buffalo, which is before circulating antigens can be detected (8). Diagnostic antigens, including *Fg*ESP, *Fg*F22, r*Fg*CatL1, and r*Fg*SAP-2, have been widely used in recent years for the establishment of ELISAs, with all producing the desired effect (9–12). However, their labor-intensive and time-consuming nature, along with the professional personnel and special laboratory materials and equipment required for their use, render ELISA unsuitable for use in the field. Hence, a convenient and rapid test, such as an immunochromatographic strip, is needed for in-field diagnosis of *F. gigantica* infection in buffalo. This study pioneered a colloidal gold immunochromatographic strip based on F22 to detect antibodies against *F. gigantica* in buffalo. The specificity, sensitivity, and stability of this testing method were evaluated by *F. gigantica*-positive and -negative sera. Results were independently validated via both ELISA and the strip test.

## Materials and methods

### Sera collection

The animal study, including sera collection were approved by the Ethics Committee of the School of Animal Science and Technology, Guangxi University. The animals used in this study were handled in accordance with good animal practices as required by the Animal Ethics Procedures and Guidelines of the People's Republic of China.

All sera employed in this study were buffalo-derived. Reference sera, including *F. gigantica*-positive and -negative sera, the positive serum was collected from buffaloes artificially infected with 200 metacercariaes 4 weeks post-infection, and the negative serum was provided by Guangxi Buffalo Research Institute, Chinese Academy of Agricultural Sciences. Sera positive for *Schistosoma japonicum* and *Babesia bovis* were kindly provided by the Shanghai Veterinary Research Institute, Chinese Academy of Agricultural Sciences and the Lanzhou Veterinary Research Institute. A total of 17 *F. gigantica* positive sera were used for detection, including 11 fluke-positive sera and 6 experiment-positive sera. Fluke-positive sera were collected from a slaughterhouse in Nanning City, Guangxi Zhuang Autonomous Region. Six experimentally-infected sera were collected from buffaloes artificially infected with 250 metacercariaes. Samples of *F. gigantica*-infected serum (250 metacercaria infected) 0–14 weeks pi were collected weekly, and buffaloes were tested *Fasciola* infection-negative before the infection. Adult flukes of *Fasciola gigantica* were found in liver and bile duct of the infected buffaloes after autopsy and eggs were also found in the feces of the artificially infected buffaloes, which confirmed that the sera from these buffaloes were positive. The Buffalo Institute of Guangxi Zhuang Autonomous Region kindly provided 325 untested sera, of which 100 were selected randomly for further diagnosis. These buffaloes provided with sera were treated by adding Niclofolan (5 mg/kg) to the feed grain, once in March and once in October every year.

### Preparation of antigen F22

*Fasciola gigantica* flukes were collected and Excretory-Secretory Product (*Fg*ESP) then collected. Flukes were washed with 37°C pre-warmed PBS 3 to 4 times and 0.01 M PBS (filtered via 0.22 μm filter) was added into the petri dish containing washed flukes (2 flukes/mL), which was then incubated at 37°C for 2 h. The culture broth was centrifuged at 3,000 *g* for 30 min and the supernatant collected after filtration with a 0.45 μm filter (Millipore, USA).

F22 was prepared as described by Jin (11). Briefly, the concentration of *Fg*ESP was adjusted to 10 mg/mL after lyophilization for subsequent chromatography. The column (GE Healthcare, USA) was equilibrated with 0.01 M PBS and 5 mL of *Fg*ESP was loaded. The protein was eluted with 0.01M PBS, the volume of each component was 2 mL, and the 22^nd^ component was collected. A bicinchoninic acid (BCA) Protein Assay Kit (TIANGEN BIOTECH, Beijing, China) was then applied to determine protein concentration.

### Preparation of 40 nm colloidal gold

Colloidal gold was prepared according to published methods (13). Briefly, 1 mL of 1% trisodium citrate (w/v) was quickly added to 100 mL of 0.01% HAuCl_4_ solution (w/v), heated to a slight boil, and stirred constantly for 15 min. As the solution naturally cooled to room temperature (RT), the pH was adjusted to 7.3 using 0.01 M potassium carbonate.

### Preparation and labeling of 2G7 colloidal gold

Mouse anti-buffalo secondary antibody 2G7 was prepared according to the Wu method (14). Briefly, to thaw the frozen hybridoma cell strain, vials were quickly warmed in a 37°C water bath and gently washed with 10 mL of pre-warmed DMEM medium. Broth containing hybridomas was centrifuged at 1,000 rpm and resuspended with complete medium containing 20% fetal bovine serum. Hybridoma cells were cultured in a 37°C germ-free incubator containing 5% CO_2_ for 24 h until they were adhered to the wall, with replacement of one half of the complete medium; these were then used to expand the culture and produce sufficient hybridoma cells. To obtain the monoclonal antibody 2G7, 0.2 mL hybridoma cells with the concentration 3 × 10^6^/mL were intraperitoneally injected into a total of 10 mice of 8-week-old female BALB/c. One week after injection, the ascites fluid was collected and centrifuged at 12,000 rpm for 20 min; supernatant with high titer was then collected and stored at −80°C for later use.

Then 18 μL of 2G7 mouse anti-buffalo secondary antibody (1 mg/mL) was added to 1 mL of colloidal gold solution and shaken gently for 15 min, after which 0.1 mL 10% BSA (filtered via 0.45 μm filter) was added to block non-specific binding sites. The resulting solution was centrifuged at 12,000 rpm for 30 min and the resuspended pellet in 0.2 mL of resuspension buffer (0.05 M sodium borate, 5% BSA, 20% sucrose, 0.1% Tween-20) was sprayed onto glass fiber pads and dried at 37°C for 90 min.

### Assembly of the immunochromatographic strip

Nitrocellulose membrane was incubated with F22 at the test line (T line) and incubated with goat anti-mouse secondary antibody at the control line (C line). Incubated membranes were then dried at 37°C for 30 min. The sample pad, conjugate pad, nitrocellulose membrane, and absorbent pad were subsequently assembled on a backing plate in the appropriate order. Then assembled plate was cut into 4 mm × 80 mm strips using a cutting machine.

### Detection of sensitivity, specificity, and stability

For detection limit study of *Fg*ICS, the reference *F. gigantica*-positive serum was gradient diluted, including 1:4, 1:16, 1:64, 1:128, 1:256, 1:1,024, and 1:4,096 dilutions. These diluted sera were dispensed onto the sample pad with 100 μL of each test strip and the results observed after 10 min. The detection limit study of indirect ELISA based on *Fg*ESP were also performed.

For cross reactivity study, 10 sera positive for *S. japonicum* and 1 serum positive for *B. bovis* were tested with a dilution of 1:64, performed as described above. The cross reactivity study of indirect ELISA based on *Fg*ESP were also performed.

For stability detection, prepared test strips were sealed together with a desiccant and stored at 4°C. After being stored for 1 and 3 months, test strips of the same batch were applied to detect the reference *F. gigantica*-positive and *F. gigantica*-negative sera.

### Early diagnosis effect evaluation

To evaluate the early diagnostic efficiency of the *Fg*ICS, sera of 3 *F. gigantica*-infected buffalo collected before the infection (week 0th) and 1–14 weeks pi weekly were tested. An aliquot of 100 μL of each serum with the serial dilution 1:64 was dispensed onto the sample pad, and the earliest week post-infection of each buffalo when antibodies could be detected was confirmed with a positive result via ICS. Indirect ELISA based on *Fg*ESP was also performed on these sera according to methods developed by Zhang et al. (15), with some modifications to make a comparison between these two methods.

### Diagnosis of *F. gigantica*

The *Fg*ICS strips and indirect ELISA based on *Fg*ESP were used in parallel for the detection of *F. gigantica* antibodies in buffalo. Of the 117 sera administered for subsequent diagnosis, 17 were positive and 100 were untested. Both *Fg*ICS and ELISA were performed as described above.

The agreement of positive serum rates between *Fg*ICS and *Fg*ESP-ELISA was evaluated using Cohen's Kappa (*k*) statistic (SPSS version 26), with agreement considered almost perfect (0.8 < *k* < 1), substantial (0.6 < *k* < 0.8), moderate (0.4 < *k* < 0.6), fair (0.2 < *k* < 0.4), or slight (0 < *k* < 0.2).

## Results

### F22 preparation

**Figure 1:**
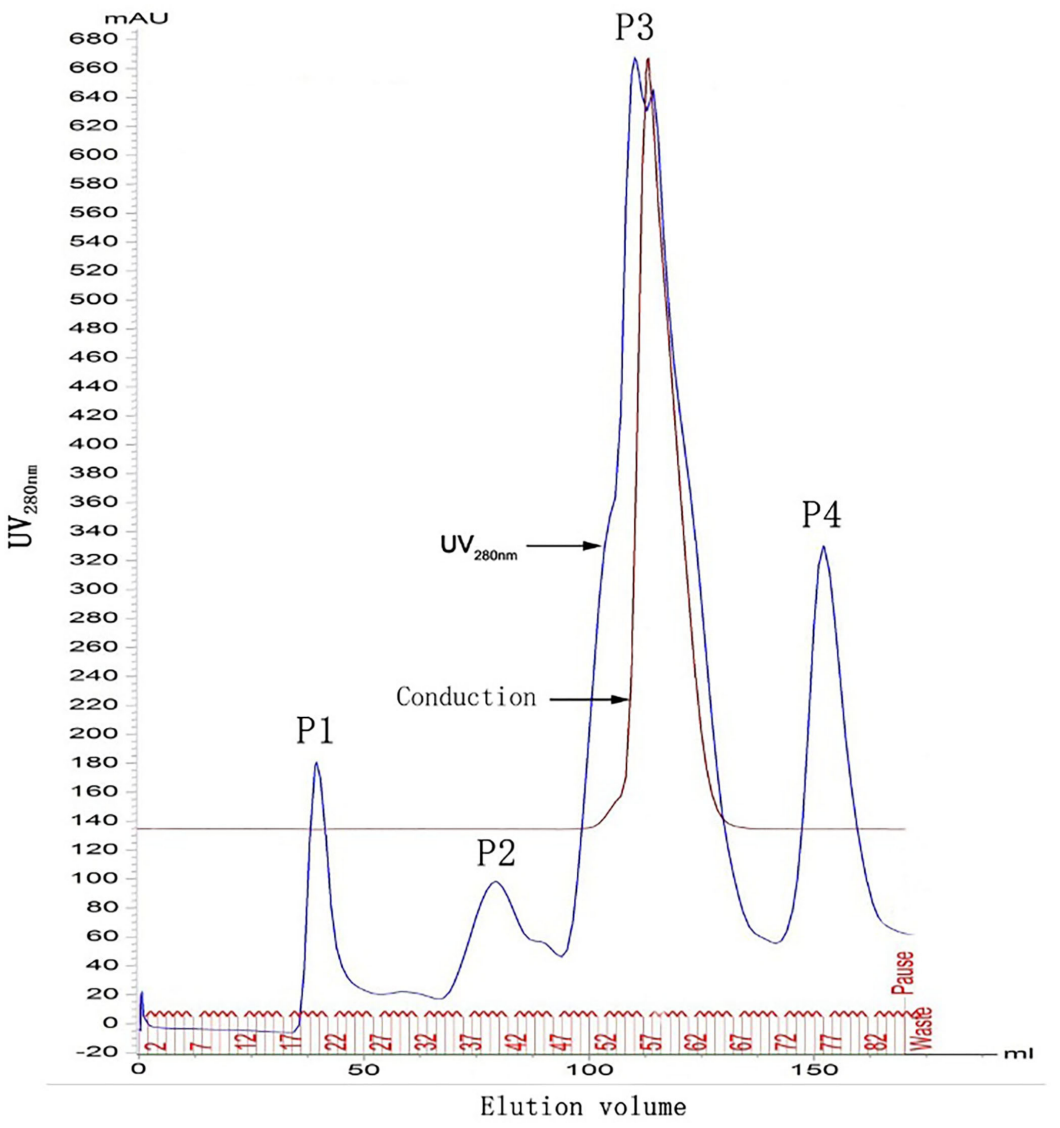
Chromatographic graphs of *Fg*ESP. P1, P2, P3, and P4 indicate 4 protein absorption peaks at UV_280nm_. F22 was located in the first peak (P1).

As shown in Figure 1, four protein absorption peaks at UV_280nm_ were successively generated from chromatography. F22 was located in the first peak (P1).

### Sensitivity, specificity, and stability detection

**Figure 2:**
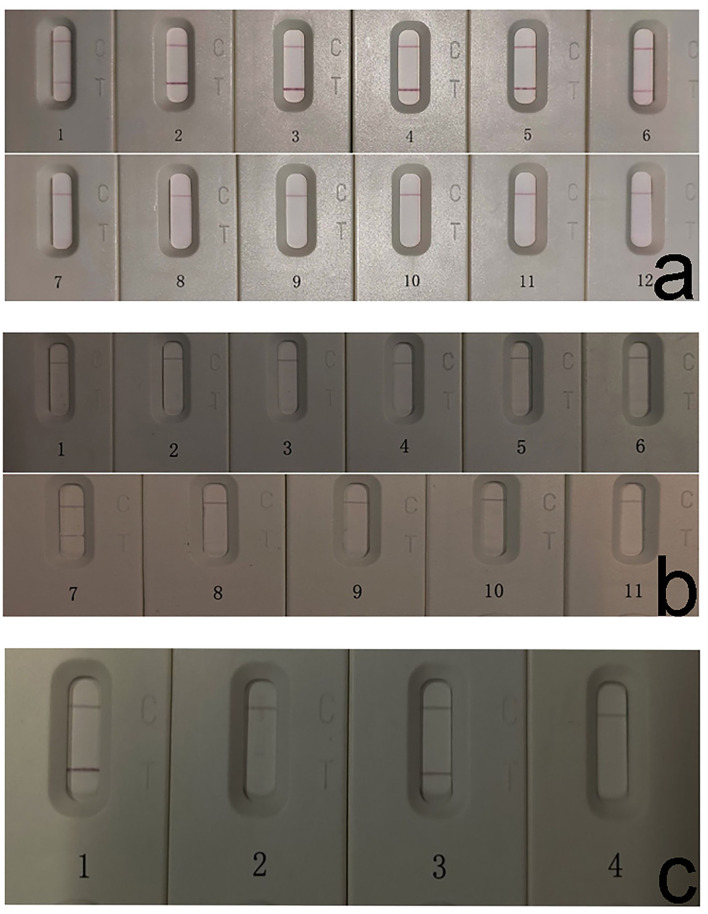
Sensitivity, specificity, and stability detection of *Fg*ICS. **(a)** Strip sensitivity detection showed low detection limit of *Fg*ICS even at 1:4096. *Fg*ICS detection limit was evaluated via serial dilution of the positive and negative sera. 1–6 (positive): 1:4, 1:16, 1:64, 1:256, 1:1,024, 1:4,096; 7–12 (negative): 1:4, 1:16, 1:64, 1:256, 1:1,024, 1:4,096. The test line result can be judged by the naked eye. **(b)** Strip specificity tests showed no cross-reactivity except for one serum positive for *Schistosoma japonicum*. Buffalo sera positive for *S. japonicum* and *B. bovis* were used to determine strip test specificity. 1–10: *S. japonicum*-positive sera. 11: *Babesia bovis*-positive serum. **(c)** Strip was stable after storage for up to 3 months. 1–2: The stability of the *Fg*ICS after storage for 1 month; 3–4: The stability of the *Fg*ICS after storage for 3 months. While 1 and 3 were reference *F. gigantica*-positive serum, 2 and 4 were *F. gigantica*-negative serum.

The prepared strips were applied to detect different dilutions of reference sera. Positive sera provided a visible color at both the T line and C line of the strip even when diluted 4,096 times. It was observed that when the serum dilution factor is >1:16 or <1:1,024, the red color of the T-line becomes significantly lighter (Figure 2a). Negative sera appeared only at the C line, and the T line was invisible across all diluted gradients. These results indicate that the detection limit of the test strip was >1:4,096 (Figure 2a). While *Fg*ESP-ELISA can produce positive results when the reference serum is diluted 1,600 times (not shown).

Ten positive sera of *S. japonicum* and 1 positive serum of *B. bovis* were employed to evaluate the cross reactivity of the *Fg*ICS. All samples, except for one *S. japonicum*-positive serum (No. 7), yielded negative results (Figure 2b). For *Fg*ESP-ELISA, all samples, except for three *S. japonicum*-positive sera (No. 4, No. 5, and No. 7), yielded negative results (not shown).

The storage test using reference-positive and -negative sera showed that *Fg*ICS was still viable after storage at 4°C for 1 or 3 months (Figure 2c).

### Early diagnosis effect evaluation

**Figure 3:**
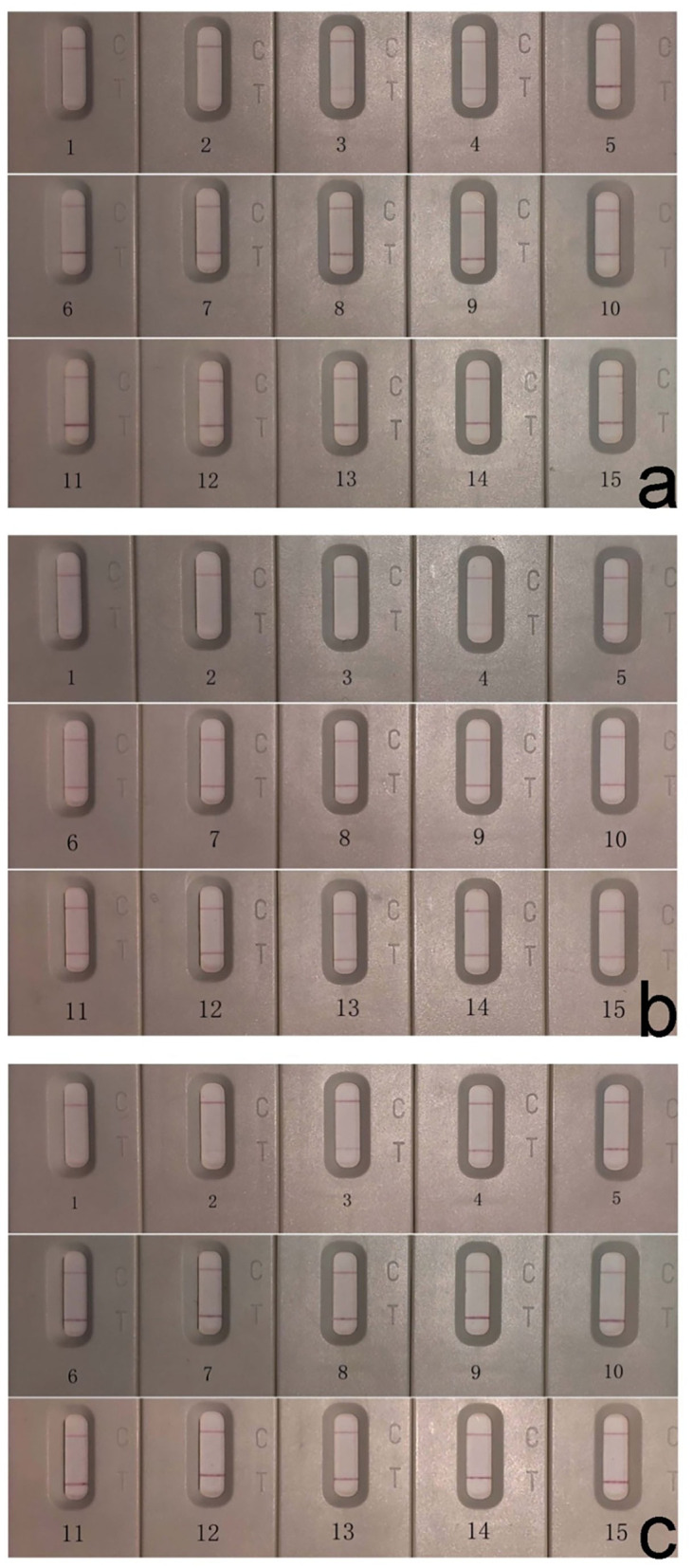
Early diagnosis effect evaluation of *Fg*ICS showed antibodies could be detected within 2–4 weeks pi. Detection of sera from 3 experimentally infected buffalo A1 **(a)**, A2 **(b)**, and A3 **(c)** by *Fg*ICS. 1–15: sera collected weekly from 0 weeks before infection and 1–14 weeks pi.

Sera of 3 buffalo experimentally infected with *F. gigantica* (A1, A2, and A3) were collected weekly from 0 to 14 weeks pi and tested using *Fg*ICS. Results showed that anti-*F. gigantica* antibodies could be detected within 2–4 weeks pi (Figure 3), just like the indirect ELISA based on *Fg*ESP (not shown).

### Diagnosis of *F. gigantica*

For 17 *F. gigantica* positive sera, all 11 fluke-positive sera tested positive via *Fg*ESP-ELISA, while 2 tested negative via *Fg*ICS (No. 2 and No. 7). Of the 6 experimentally *F. gigantica*-infected sera, all tested positive via *Fg*ESP-ELISA, while 1 tested negative via *Fg*ICS (No. 3). These results yielded positive rates of 82.4% (14/17) and 100.0% (17/17) for *Fg*ICS and ELISA, respectively (Supplementary Table 1).

*Fg*ICS and *Fg*ESP-ELISA were also applied to 100 field sera from standardly raised buffalo. The results showed that they were all negative both in *Fg*ICS and *Fg*ESP-ELISA antibody detection. The false-positive rate was 0%, and an almost perfect agreement was confirmed between *Fg*ICS and *Fg*ESP-ELISA, with a high Cohen's kappa value (*k* = 0.869).

## Discussion

Immunochromatographic strips represent a portable, rapid, and easy-to-conduct method for the seroepidemiological screening and have been widely applied in the diagnosis of parasitic infections, such as *Babesia* (16–18), *Toxoplasma* (19), *Paragonimiasis Skrjabini* (20) and *Schistosoma japonica* (21, 22). However, immunochromatographic strips in the diagnosis of *F. gigantica* did not established. Given that the antigen-antibody binding of ICS should be completed in a relatively short time (typically within 10 min), this method necessitates higher sensitivity of diagnostic antigens. For *F. gigantica*, the indirect ELISA was established based on several antigens (9–12, 23), including *Fg*ESP, r*Fg*CatL1, *Fg*F22, and r*Fg*SAP-2. Among them, *Fg*ESP and *Fg*F22 showed no cross-reactivity with cattle-derived *Paramphistomum epiclitum*-positive serum and buffalo-derived *B. bovis*-positive serum, indicating its low cross-reactivity. However, additional research that employed *Fg*ESP and *Fg*CatL1 for the development of ICS yielded no promising results. F22, the optimal component screened out from *Fg*ESP after molecular sieve chromatography, has demonstrated significantly higher sensitivity than that of *Fg*ESP in the indirect ELISA (11) and was thus selected for ICS development. Here, an immunochromatographic strip based on F22 was established. It was observed that when the serum dilution factor is >1:16 or <1:1,024, the red color of the T-line becomes significantly lighter (Figure 2). Therefore, while detecting serum via *Fg*ICS, a serum dilution ratio between 1:16 and 1:1,024 is recommended to achieve credible results. Additionally, compared with the *Fg*ESP-ELISA, *Fg*ICS showed lower detection limit of serum. This lower detection limit makes *Fg*ICS viable for fascioliasis diagnosis. Regarding the specificity detection, due to the limitations of buffalo-derived serum, only 1 sample of *B. bovis*-positive serum was applied here, and undoubtedly, more than 3 sample would convincing. Besides, considering the distant relatives between *F. gigantica* and *B. Bovis*, trematode serum such as buffalo-derived *P. epiclitum*, which causes great loss in Asian buffalo breeding, should be applied in the following cross-reactivity detection.

Results suggested that the *Fg*ICS was developed successfully. Specifically, (i) it is sensitive enough both for early detection and for latent infections; (ii) it is specific enough to differentiate between *F. gigantica* and *S. japonicum*, though its specificity must be further verified due to limited serum species; and (iii), it showed an almost perfect agreement with *Fg*ESP-ELISA. Another advantage of *Fg*ICS is that it requires no special expertise or equipment. It is also a time-saving process, needing only 10 min to be completed. Furthermore, *Fg*ICS is stable during long storage. Thus, *Fg*ICS represents a suitable diagnostic tool for the rapid detection of *F. gigantica* infection under field conditions in buffalo and for the seroepidemiological screening of buffaloes from different area, which would in turn conduce to the prevention and control of this disease.

## Conclusion

An immunochromatographic strip was developed and optimized. With relatively high sensitivity and specificity, *Fg*ICS represents a portable, reliable, and fast diagnostic tool of *F. gigantica* and was thus proposed as a powerful supplement to current diagnostic assays.

## Data availability statement

The original contributions presented in the study are included in the article/Supplementary material, further inquiries can be directed to the corresponding authors.

## Ethics statement

The animal study was reviewed and approved by Ethics Committee of the School of Animal Science and Technology, Guangxi University.

## Author contributions

WZ conceived the project. WJ carried out laboratory work. WW prepared 2G7. JW, KH, and WJ wrote the manuscript. ZW and YG performed buffalo maintenance and serum collection. WD received the manuscript and contributed to the final submission. All authors read and approved the final manuscript.

## Funding

This study was supported by the National Key Research and Development Program (Grant No. 2017YFD0501202-1) and the Science and Technology Major Project of Guangxi (Grant No. AA17204057).

## Conflict of interest

The authors declare that the research was conducted in the absence of any commercial or financial relationships that could be construed as a potential conflict of interest.

## Publisher's note

All claims expressed in this article are solely those of the authors and do not necessarily represent those of their affiliated organizations, or those of the publisher, the editors and the reviewers. Any product that may be evaluated in this article, or claim that may be made by its manufacturer, is not guaranteed or endorsed by the publisher.
