# Supplementary material for: Development of a colloidal gold immunochromatographic strip for the rapid detection of antibodies against Fasciola gigantica in buffalo
Source: Front Vet Sci. 2022 Sep 16;9:1004932. doi: 10.3389/fvets.2022.1004932 (PMC9523912; doi:10.3389/fvets.2022.1004932)
Supplement: Supplementary file 1 [file Table_1.DOCX]

Table 1 Detection of 20 serums from *F. gigantica*-infected buffaloes by *Fg*ICS and ELISA.

| Serum samples | *F. gigantica* detected in livers | | | | | | | | | | | experimentally infected with *F. gigantica* metacercaria | | | | | | Positive rate (%) |
| --- | --- | --- | --- | --- | --- | --- | --- | --- | --- | --- | --- | --- | --- | --- | --- | --- | --- | --- |
| No. | 1 | **2** | 3 | 4 | 5 | 6 | **7** | 8 | 9 | 10 | 11 | 1 | 2 | **3** | 4 | 5 | 6 |  |
| *Fg*ICS | + | **–** | + | + | + | + | **–** | + | + | + | + | + | + | **–** | + | + | + | 82.4 |
| ELISA | + | **+** | + | + | + | + | **+** | + | + | + | + | + | + | **+** | + | + | + | 100.0 |

+, positive; –, negative
